# Pregnant women co-infected with HIV and Zika: Outcomes and birth defects in infants according to maternal symptomatology

**DOI:** 10.1371/journal.pone.0200168

**Published:** 2018-07-06

**Authors:** Esaú C. João, Orlando da C. Ferreira, Maria Isabel Gouvêa, Maria de Lourdes B. Teixeira, Amilcar Tanuri, Luiza M. Higa, Deise A. Costa, Ronaldo Mohana-Borges, Mônica B. Arruda, Haroldo J. Matos, Maria Leticia Cruz, Wallace Mendes-Silva, Jennifer S. Read

**Affiliations:** 1 Rio de Janeiro, RJ, Brazil—Infectious Diseases Department, Hospital Federal dos Servidores do Estado, Rio de Janeiro, Rio de Janeiro, Brazil; 2 Laboratório de Biologia Molecular, Departamento de Genética, Instituto de Biologia, Universidade Federal do Rio de Janeiro—Rio de Janeiro, Rio de Janeiro, Brazil; 3 Instituto Nacional de Infectologia Evandro Chagas—Fundação Oswaldo Cruz, Rio de Janeiro, RJ, Brazil; 4 Laboratório de Genômica Estrutural, Instituto de Biofísica Carlos Chagas Filho, Universidade Federal do Rio de Janeiro, Rio de Janeiro, Rio de Janeiro, Brazil; 5 Departamento de Epidemiologia, Instituto Evandro Chagas, Belém, Pará, Brazil; 6 Maternal-Fetal Unit, Hospital Federal dos Servidores do Estado, Rio de Janeiro, Rio de Janeiro, Brazil; 7 Department of Epidemiology and Biostatistics, University of California at San Francisco, San Francisco, California, United States of America; University of Nebraska Medical Center, UNITED STATES

## Abstract

**Background:**

Zika virus (ZIKV) was first isolated in Uganda in 1947. In Brazil, the first reported case of ZIKV infection was in May 2015. Additionally, dengue (DENV) is endemic and there has been a recent outbreak of chikungunya (CHIKV). Since the clinical manifestations of different arboviral infections (AI) can be similar, definitive diagnosis requires laboratory testing.

**Objectives:**

To determine the prevalence of ZIKV, DENV, and CHIKV infections in a Brazilian cohort of HIV-infected pregnant women, to assess clinical/immunological characteristics and pregnancy outcomes of women with evidence of recent AI.

**Study design:**

Laboratory diagnosis of ZIKV, DENV and CHIKV infections utilized serological assays, RT-PCR and PRNT. The tests were performed at the first visit, 34–36 weeks of gestation and at any time if a woman had symptoms suggestive of AI. Mann-Whitney tests were used for comparison of medians, Chi-square or Fisher’s to compare proportions; *p*< 0.05 was considered statistically significant. Poisson regression was used to analyze risk factors for central nervous system (CNS) malformations in the infant according to maternal symptomatology.

**Results:**

Of 219 HIV-infected pregnant women enrolled, 92% were DENV IgG+; 47(22%) had laboratory evidence of recent AI. Of these, 34 (72%) were ZIKV+, nine (19%) CHIKV+, and two (4%) DENV+. Symptoms consistent with AI were observed in 23 (10%) women, of whom 10 (43%) were ZIKV+, eight (35%) CHIKV+. No CNS abnormalities were observed among infants of DENV+ or CHIKV+ women; four infants with CNS abnormalities were born to ZIKV+ women (three symptomatic). Infants born to ZIKV+ women had a higher risk of CNS malformations if the mother was symptomatic (RR = 7.20), albeit not statistically significant (*p* = 0.066).

**Conclusions:**

Among HIV-infected pregnant women with laboratory evidence of a recent AI, 72% were ZIKV-infected. In this cohort, CNS malformations occurred among infants born to both symptomatic and asymptomatic pregnant women with Zika infection.

## Introduction

Zika virus (ZIKV) is an arbovirus of the genus Flavivirus and was first isolated from a Rhesus monkey in the Zika forest of Uganda in 1947 [1]. The first human cases were described in Nigeria in 1954, and subsequently sporadic cases have been described in humans [1,2]. In Brazil, the first case of ZIKV infection was reported in May 2015 and since then ZIKV infection has affected thousands of individuals, initially in the northeast and eventually spreading throughout the country [3,4].

In the Americas, the most important vectors are *Aedes aegypti* and *Aedes albopictus*, which can also transmit other arboviruses such as dengue virus (DENV) and chikungunya virus (CHIKV) [5]. Mother-to-child transmission of ZIKV can occur during the pregnancy [6], and recently Blohm published a case report of possible transmission of ZIKV through breast milk [7].

In Brazil, DENV is endemic and there has been a recent outbreak of CHIKV [8]. Individuals infected with ZIKV and other arboviruses frequently present with similar clinical features, making laboratory testing an important component of the diagnostic process [9]. However, antibody cross-reactivity between DENV and ZIKV hampers serological distinction of these infections [4]. Molecular tests for ZIKV, DENV and CHIKV are constrained by the short duration of viremia (and/or viruria) and the limited capacity of laboratories to perform molecular assays [4,10].

A large proportion of ZIKV infections are asymptomatic [4]. If symptomatic, the most frequent signs and symptoms are rash, with or without fever, pruritus, arthralgia, myalgia, conjunctivitis and fatigue [4]. In adults, the most serious complication of ZIKV infection is Guillain-Barré Syndrome [11]. Pregnant women infected with ZIKV may have adverse pregnancy outcomes, including abortion, stillbirth, or infants born preterm and/or with the Congenital Zika Syndrome, characterized by microcephaly, cerebral calcifications, ventriculomegaly, and arthrogryposis [12].

In Brazil, there are nearly 900,000 HIV-infected individuals [13] and 231,725,cases of suspected ZIKV infection were reported by the Pan American Health Organization [14]. Nevertheless, there are few reports of ZIKV and HIV coinfection [15,16]. More specifically, there are limited data regarding ZIKV infection in HIV-infected pregnant women and the consequences of such coinfection in these women and their infants. In addition, there is no evidence regarding whether ZIKV infection could worsen HIV infection and *vice versa* [17].

The aims of this study were to determine the prevalence of ZIKV, CHIKV and DENV infection in a Brazilian cohort of HIV-infected pregnant women, and to assess pregnancy outcomes of women with evidence of recent arboviral infections.

## Materials and methods

The study population comprised HIV-infected pregnant women and their infants at a referral center for prevention of mother-to-child transmission of HIV in Rio de Janeiro, Brazil from January 2015 to August 2016 [18]. As part of routine care at the center, all the pregnant women were using antiretrovirals (ARVs) for prevention of mother-to-child transmission and none of their infants was breastfed. A medical history was obtained and a physical examination was performed at each clinical visit. At cohort entry, the following laboratory tests were performed: Treponemal and non-treponemal tests, *Toxoplasma* IgM and IgG, cytomegalovirus IgM and IgG, rubella IgM and IgG, hepatitis B surface antigen, and antibodies against hepatitis A, B and C. All women also underwent regular ultrasonography evaluation as part of routine prenatal care (once each trimester) and at any other time when indicated, using a 3.5MHz curved array probe, Philips HD11 XE (Philips Healthcare, Royal Philips Electronics, Eindhoven, The Netherlands). Diagnostic tests for arboviral infection (see below) were performed at the first prenatal visit, at 34–36 weeks of gestation, and at any time if a woman had symptoms suggestive of arbovirus infection (maculopapular rash, pruritus, fever, headache, retro-orbital pain, photophobia, arthralgia, arthritis, myalgia, fatigue, conjunctivitis). Microcephaly was defined according to Centers for Disease Control and Prevention (CDC) criteria by measurement of occipitofrontal circumference, based on standard growth charts for sex, age and gestational age at birth (among term neonates, defined as ≤ 31.9 centimeters for boys and ≤31.5 centimeters for girls; Fenton criteria were used for preterm infants) [19]. Preterm birth was defined as a Capurro index of less than 37 weeks gestation at birth.

All asymptomatic women underwent screening for ZIKV, DENV, and CHIKV antibodies (IgM and IgG). The following serological assays were utilized for detection of ZIKV IgM and IgG antibodies: The *Dual Path Platform (DPP) ZIKV IgM/IgG rapid test* (Chembio Diagnostic Systems, Inc., Medford, New York, USA), *Anti-ZIKV ELISA IgM and IgG* (Euroimmun, Luebeck, Germany) and *Anti-ZIKV MAC-ELISA IgM* (CDC, US). DENV IgM and IgG antibodies were assayed by *DENV-specific IgM capture ELISA and IgG indirect ELISA* (Standard Diagnostic, Inc, Gyeonggi-do, Korea). CHIKV IgM and IgG antibodies were detected by *CHIKV IgM and IgG ELISA* (Euroimmun, Luebeck, Germany).

The serological algorithms to diagnose ZIKV and DENV infections interdigitate but are described separately. The algorithm for Zika uses two parallel tests to detect for both ZIKV IgM and ZIKV IgG (DPP rapid test and EuroImmun ELISA). In the absence of a positive ZIKV reverse transcription polymerase chain reaction (RT-PCR) result (see below), samples with negative results in all these four assay parameters were considered to have no evidence of recent ZIKV infection. Samples with any combination of positive serological test results for ZIKV IgM or IgG were then evaluated with CDC MAC ELISA for ZIKV. Samples with reactivity to ZIKV IgM on the CDC MAC ELISA were then assayed by the plaque reduction neutralization test (PRNT), for both ZIKV and DENV.

The diagnostic algorithm for DENV is simpler. Samples are initially screened for the presence of IgM antibodies to DENV. IgM-positive samples are further tested by the PRNT, for both ZIKV and DENV.

**Fig 1:**
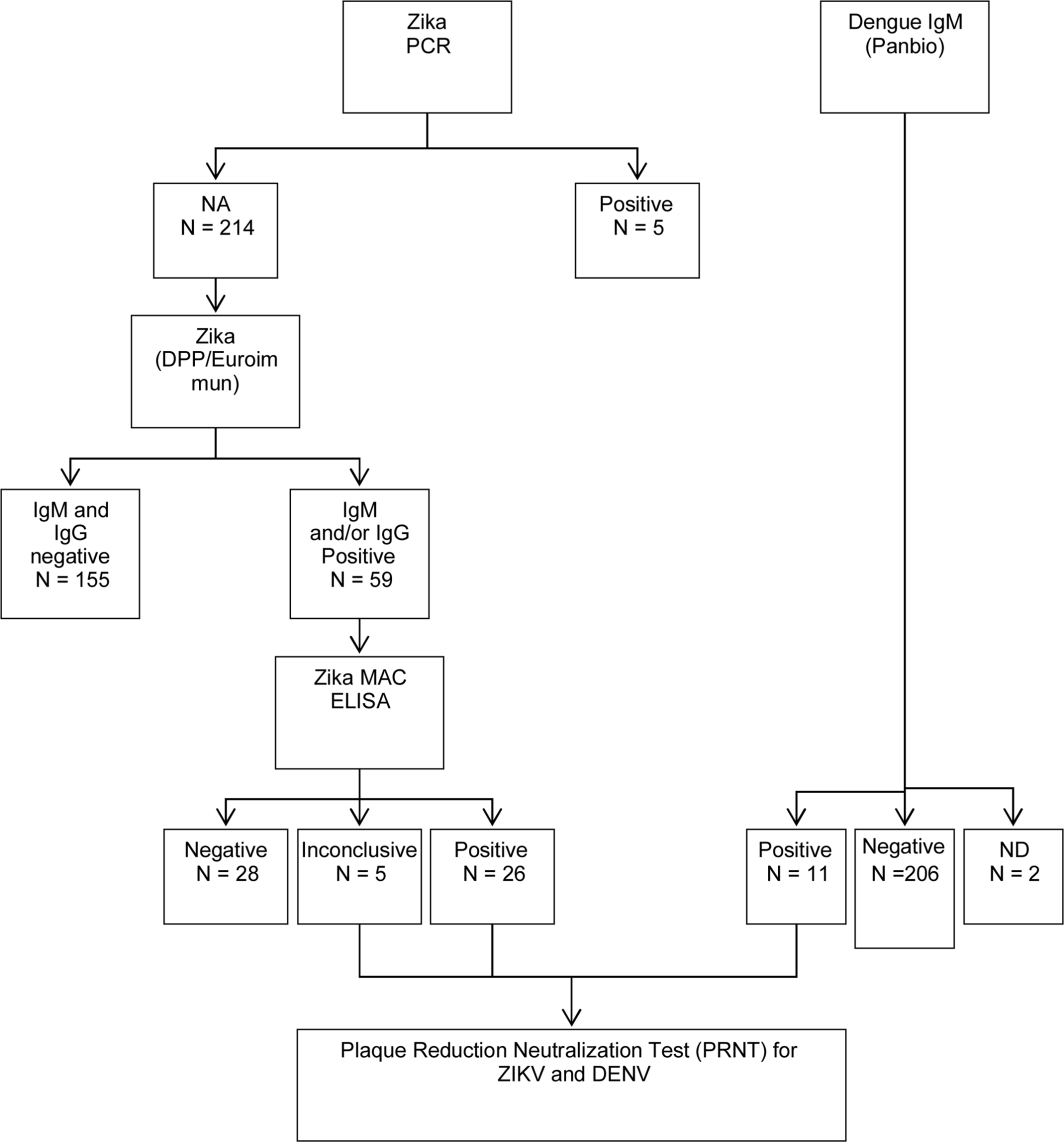
Algorithm for diagnostic tests in symptomatic and asymptomatic pregnant women.

The final interpretation took into consideration both the presence of ZIKV- and/or DENV-specific IgM antibodies as well as the antibody titers against ZIKV and DENV on the PRNT. This interpretation follows closely the CDC recommendations for the interpretation of these tests [20] (Fig 1).

These women were also screened for IgM and IgG antibodies against CHIKV. The criteria for definition of CHIKV infection were: IgG seroconversion during pregnancy or the presence of IgM antibody, and a CHIKV PRNT titer greater than 1/20.

The same diagnostic protocol was applied in the case of asymptomatic women, with the exception that RT-PCR was never performed due to the absence of signs and symptoms of acute infection.

Molecular assays for detection of ZIKV and CHIKV RNA by real-time RT-PCR were performed as described [21–23]. These assays were performed whenever the specimen (plasma, urine or both) was collected within the time frame of PCR detectability (within 14 days of symptom onset). A RT-PCR positive result for any of these viruses indicates recent infection for this particular virus. The cutoff value for threshold cycles was 38 for all testing parameters.

PRNTs against ZIKV, CHIKV and DENV were carried out according to the study algorithm and performed as described [20]. The PRNT detects the presence of neutralizing antibodies to these three viruses, thereby helping to evaluate the specificity of the serological reactivity to ZIKV and to confirm the diagnosis of CHIKV infection. A PRNT result greater than 1/20 was considered positive.

Mann-Whitney tests were used for comparison of medians, and interquartile range (IQR) intervals were also calculated. Chi-square or Fisher’s exact tests were used to compare proportions and 95% confidence intervals (CIs) were estimated for these proportions. All *p* values were two-sided with *p*< 0.05 being considered statistically significant. The risk ratio for CNS malformations in the infant between symptomatic and asymptomatic pregnant women was calculated with the 95% CI estimated by Taylor’s series. A multivariate Poisson model was used to analyze risk factors for CNS malformations among the HIV/ZIKV co-infected mothers. R software (version 3.3) was used to perform modelling [24,25].

The study was approved by the Human Research Ethics Committee of Hospital Federal dos Servidores do Estado (study approval number 1.539.650) and written inform consent was obtained.

## Results

Between January 2015 and August 2016, a period of high risk for ZIKV infection in Rio de Janeiro, 219 HIV-infected pregnant women enrolled in the cohort. Characteristics of the 219 women overall are shown in Table 1. After enrollment, 23 pregnant women had symptoms consistent with arboviral infection. The main symptoms reported were rash, arthralgia/arthritis, and pruritus. Fever, myalgia, fatigue, headache, conjunctivitis and nausea were also described.

**Table 1:** Characteristics of HIV-infected pregnant women, overall and according to ZIKV co-infection.

Characteristic	Total (N = 219)		ZIKV infection		No ZIKV infection		*p-*value
			(N = 34)		(N = 185)		
	N	%	N	%	N	%	
**Age (years)**	**219**		**34**		**185**		0.77
<20	29	13.24	4	11.80	25	13.50	
20–29	123	56.16	18	52.90	105	56.80	
30–39	60	27.40	10	29.40	50	27.00	
≥40	7	3.20	2	5.90	5	2.700	
**Ethnicity**	**219**		**34**		**185**		0.23
White	58	26.48	12	35.20	46	24.90	
Black	62	28.31	11	32.40	51	27.60	
Mixed	99	45.21	11	32.40	88	47.50	
**Marital status**	**199**		**34**		**185**		0.39
Single	80	36.53	17	50.00	63	34.10	
Married	18	8.22	1	2.90	17	9.20	
Stable union	99	54.34	16	47.10	103	55.70	
Divorced	1	0.46	-	-	1	0.50	
Widowed	1	0.46	-	-	1	0.50	
**Education (years)**	**219**		**34**		**185**		0.75
0–4	19	8.68	4	11.80	15	8.10	
5–9	98	44.75	15	44.10	83	44.90	
10–14	98	44.75	15	44.10	83	44.90	
>15	4	4.00	-	-	4	2.10	
**CD4 count (cells/mm**^**3**^**) at enrollment**	**219**		**34**		**185**		0.32
< 200	29	13.25	2	5.9	27	14.16	
≥ 200, <500	98	44.75	15	44.10	83	44.90	
≥ 500	92	42.00	17	50.00	75	40.50	
**Detectable HIV viral load (copies/mL) at enrollment**	**219**		**34**		**185**		0.13
Yes	170	77.63	23	67.60	147	79.50	
No	49	22.37	11	32.40	38	20.50	
**Detectable HIV viral load (copies/mL) near delivery**	**210**		**32**		**178**		0.14
Yes	77	36.77	8	85.00	69	38.80	
No	133	63.33	24	75.00	109	61.20	
**Timing of ARV start**	**219**		**34**		**185**		0.02
Before conception	79	36.07	18	52.94	61	32.97	
During pregnancy	140	63.93	16	47.06	124	67.03	
**Symptoms consistent with arboviral infection**	**219**		**34**		**185**		0.0012
Yes	23	10.50	10	29.4	13	7.0	
No	196	89.50	24	70.6	72	93.0.	

All 219 pregnant women (196 asymptomatic and 23 symptomatic) had at least one blood sample collected for arboviral infection diagnosis, and at least two samples were collected from 197 (91%) of women. Among the symptomatic pregnant women, 11 urine samples were also collected. The median time interval between the first and second blood sample was 79 days (IQR 36–173) for symptomatic women and 123 days (IQR 77–167) for asymptomatic women. Of all HIV-infected pregnant women enrolled, 201/219 (92%) were DENV IgG positive and 47/219 (22%) had laboratory evidence of possible arboviral infection.

Of these 47 women, 13 had evidence of recent ZIKV infection, two of recent DENV infection, nine of recent CHIKV infection, two had inconclusive results and another 21 women had evidence of Flavivirus infection, not otherwise specified. See Table 2.

**Table 2:** Interpretation of ZIKV and DENV laboratory assays.

Zika PCR	Zika IgM / IgG	Zika IgM	Dengue IgM	Zika PRNT	Dengue PRNT	# of cases	Interpretation
Positive	-	-	-	-	-	5	Confirmed Zika virus infection
-	Positive	Positive	Negative	>20	≤20	6	Recent Zika virus infection
-	Positive	Positive	Positive	>20	≤20	1	Recent Zika virus infection
-	Positive	Positive	Negative	>20	>20	14	Recent Flavivirus infection
-	Positive	Positive	Positive	>20	>20	5	Recent Flavivirus infection
-	Positive	Inconclusive	Negative	>20	≤20	1	Evidence Zika virus infection
-	Positive	Inconclusive	Negative	>20	>20	2	Evidence Flavivirus infection
-	Positive	Negative	Positive	≤20	>20	1	Recent Dengue virus infection
-	Negative	Not done	Positive	≤20	>20	1	Recent Dengue virus infection
-	Positive	Inconclusive	Negative	—	—	2	Inconclusive results
-	Negative	Not done	Positive	≤20	≤20	3	No evidence of Zika or dengue virus infection
-	Positive	Negative	Negative	—	—	27	No evidence of Zika or dengue virus infection
-	Negative	Not done	Negative	—	—	151	No evidence of Zika or dengue virus infection

In this group, no serological distinction between the ZIKV and DENV infections could be made. However, for the purpose of this manuscript, these samples were classified as recent ZIKV infections on the basis of the severe ZIKV epidemic in Rio de Janeiro during the study period. Of the 34 women classified as having a recent ZIKV infection, all were CHIKV-negative. Five were ZIKV RT-PCR-positive (4 plasma samples, 1 urine and 1 plasma and urine positive) and 20 were ZIKV IgM-positive (all of these 25 women were DENV IgM-negative). Six of the remaining nine women had also DENV IgM antibodies. An additional three women were DENV IgM-negative and had an inconclusive ZIKV IgM result. In all these 34 cases classified as recent ZIKV infection, ZIKV PRNT titers were always greater than the cutoff of 1/20.

Of the two women with evidence of recent DENV infection (positive for DENV IgM and with a PRNT titer greater than 1/20), both were negative for CHIKV. Of the nine women with recent CHIKV infection, seven were CHIKV IgM-positive, one was CHIKV PCR-positive and negative for IgM, and one had both the CHIKV PCR and IgM assays positive. In all cases, CHIKV PRNT titers were greater than 1/20.

A comparison of characteristics of the study population according to the presence or absence of ZIKV infection is shown in Table 1. Characteristics of the HIV-infected women, including CD4 count and HIV viral load, did not differ according to ZIKV co-infection, except that ZIKV-infected women were more likely to have symptoms consistent with arboviral infection (p = 0.0012).

The 219 women in the study population delivered 221 live born infants, including two pairs of twins. Of the 221 live born infants, the median birth weight was 3000 grams (IQR: 2670–3340), and 12% were preterm. Two neonates (0.90%) had laboratory evidence of HIV infection, but their mothers did not have arboviral co-infection. Adverse pregnancy and infant outcomes included three miscarriages, three stillbirths, and one case of neonatal death.

Of the 219 women, four (2%) had infants with CNS abnormalities. All four of these infants were born to women with ZIKV infection (4/34, 11.76%). Of the four mothers, two were ZIKV RT-PCR-positive and two were ZIKV IgM-positive (all were DENV IgM-negative). A Poisson multivariate model with a square link function was used to analyze risk factors associated with infant CNS malformations. See Table 3. The outcome variable was the rate of CNS malformations. Variables added to the model were: maternal CD4+ count, maternal HIV viral load, use of ARVs before pregnancy, and maternal ZIKV infection. Maternal ZIKV infection was the only variable associated with CNS malformations in the infant. Additionally, no interactions between variables was observed. Residual analysis demonstrated good model fit.

**Table 3:** Results of multivariate Poisson modeling of risk factors (among 219 HIV-infected pregnant women) associated with CNS malformations in their infants.

Variables	Coefficient	Stderror	Z value	*p* -value
**Intercept**	0.1550	0.1476	1.051	0.29342
**ZIKV infection**	0.2849	0.09462	3.011	**0.00261**
**CD4 cell count/mm**^**3**^ **at enrollment**	-0.00005084	0.0001245	-0.408	0.68308
**HIV viral load at enrollment (Log)**	-0.0003098	0.01233	-0.025	0.97995
**Use of ARVs before pregnancy**	-0.04603	0.07792	-0.591	0.55469

Table 4 provides further detail regarding the four infants. Three infants were born to symptomatic mothers (3/10, 30%), of whom two presented with clinical symptoms during first trimester and one in the second trimester. One infant had an asymptomatic mother (1/24, 4.2%) with laboratory evidence of ZIKV infection between 13 and 30 weeks of gestation. Although infants of symptomatic women with ZIKV infection were more likely to have CNS malformations compared to infants born to asymptomatic women who were ZIKV-infected, this association was not statistically significant (RR = 7.20; 95%CI 0.84–61.17; p = 0.066 (Fisher’s exact test)).

**Table 4:** Characteristics of infants with CNS malformations (n = 4) born to mothers with laboratory evidence of ZIKV infection during pregnancy.

CNS Malformation	Gestational age at birth (weeks)	Gestational age at maternal ZIKV infection	Maternal CD4 cell count (cells/mm^3^)at enrollment	Maternal HIV viral load (Log10) at enrollment	Maternal HIV viral load (Log10) near delivery	Use of ARVs before pregnancy
Microcephaly, arthrogryposis, hydrops (at ultrasonography)	20 (fetal loss)	6	169	Undetectable	NA^a^	Yes
Hydrocephaly, cerebral calcifications, meningomyelocele	37	7	500	Undetectable	Undetectable	Yes
Ventriculomegaly, brain calcifications, microcephaly	40	24	262	4.3	1.77	No
MIcrocephaly	39	>12 and <32^b^	996	3.5	Undetectable	Yes

^a^ Not applicable–abortion

^b^ Asymptomatic. Seroconversion between >12 and < 32 weeks of gestation

## Discussion

In this analysis of arboviral infections among HIV-infected pregnant women in Rio de Janeiro, Brazil during the recent ZIKV epidemic, almost all had evidence of past exposure to DENV and approximately one-fifth had laboratory evidence of recent arboviral infection. Of those with a recent arboviral infection, over two-thirds were infected with ZIKV. Most women with recent arboviral infections were asymptomatic, as were those with recent ZIKV infection. All infants born with CNS abnormalities had mothers with ZIKV infection during pregnancy. Infants born to mothers with symptomatic ZIKV infection had a higher, albeit not statistically significant, risk of CNS malformations that those born to asymptomatic, ZIKV-infected mothers.

The high proportion of women who were DENV IgG-positive is consistent with previous reports from Brazil in which 88–90% of participants had evidence of previous exposure to DENV [26]. All DENV serotypes circulate and occur in several states in Brazil and, in 2016, over 1.5 million cases of DENV were reported [27]. CHIKV infections have occurred in Brazil since 2014, spreading rapidly through several regions of the country. According to the Brazilian Ministry of Health, the incidence rate of CHIKV infection in 2016 was 134,8/100,000 [27].

As has been recognized in previous studies, many women with ZIKV infection in this study were asymptomatic. However, the women in this cohort were HIV-infected, and one could hypothesize that immunodeficiency associated with HIV infection could result in more symptomatic ZIKV infections. But, 42% of the HIV-infected women had a CD4 count at or above 500 cells/mm^3^ (median CD4 count was 459 cells/mm^3^) at enrollment, and only a minority had a detectable viral load at enrollment or near delivery (although a higher proportion had a detectable viral load near delivery). Furthermore, all of the women were using ARVs during pregnancy, with 79 (36%) initiating ARVs pre-conception.

The most frequently reported signs and symptoms related to arboviral infection in this population of pregnant women were rash, arthralgia/arthritis and pruritus, similar to those described in another cohort of pregnant women with ZIKV infection in Brazil [10] and in one case report of a non-pregnant, HIV-infected individual [15]. In our study, fever was only reported in a minority of participants, similar to what was reported previously for pregnant women in Brazil [10] and Puerto Rico [28].

Severe abnormalities in the infant have been described among children born to women with ZIKV infection during pregnancy [28–30], including the abnormalities observed among the four infants with CNS abnormalities born to mothers with ZIKV infection during pregnancy in this study (microcephaly, arthrogryposis, hydrocephaly, cerebral calcifications, ventriculomegaly). Infants with CNS abnormalities were only born to women with recent ZIKV infection (and not recent DENV or CHIKV infection), but these ZIKV infections during pregnancy were not all symptomatic infections. Previously, an overall risk of congenital abnormalities in a child born to a mother with ZIKV infection during pregnancy was estimated to be approximately 5%, and it has been estimated that infants with congenital ZIKV infection were approximately equally likely to be born to a mother with symptomatic or asymptomatic ZIKV infection during pregnancy [31]. However, in our study, almost 12% of children born to women with ZIKV infection during pregnancy had CNS abnormalities, and symptomatic ZIKV infection during pregnancy was associated (although not statistically significantly) with an increased risk of such abnormalities in the infant. It remains to be seen whether this increased risk of infant CNS abnormalities, overall and possibly with symptomatic maternal infection, is confirmed in future studies of ZIKV infection in the overall population of pregnant women, or is really restricted only to infants born to HIV-infected women with ZIKV co-infection.

To the best of our knowledge, our study is the first description of arboviral infections among HIV-infected pregnant women, and the first analysis of infant outcomes of HIV-infected women with arboviral infection during pregnancy. A strength of this study is the relatively large study population and the systematic and extensive laboratory testing available for confirmation of arboviral infections among both symptomatic and asymptomatic women. However, despite the relatively large population of women enrolled in the study, there were relatively few infants born with CNS abnormalities, thus limiting our analyses of pregnancy outcomes according to arboviral infection and maternal symptomatology. In addition, laboratory diagnosis in this study, as with others conducted in areas of DENV endemicity and/or widespread yellow fever disease or immunization, remained difficult because of cross-reactivity of antibodies against different flaviviruses and limited utility of nucleic acid amplification tests (e.g., ZIKV RT-PCR) due to only transient viremia after acute infection.

## Conclusion

In summary, in this cohort of HIV-infected pregnant women with laboratory evidence of a recent arboviral infection, over two-thirds had laboratory-confirmed ZIKV infection. A relatively high proportion of infants of HIV-infected pregnant women with ZIKV co-infection during pregnancy had CNS abnormalities. In this cohort, CNS malformations occurred either in infants born to HIV infected symptomatic or asymptomatic women. Future research, involving larger study populations, should clarify these findings.

## Supporting information

S1 File Supporting information for Table 2 –interpretation of ZIKV and DENV laboratory assays. In this part, we describe all the laboratory assays performed in the population of the study.
(XLSX)
